# Maternal taurine as a modulator of Cl^–^ homeostasis as well as of glycine/GABA_A_ receptors for neocortical development

**DOI:** 10.3389/fncel.2023.1221441

**Published:** 2023-08-03

**Authors:** Tomonori Furukawa, Atsuo Fukuda

**Affiliations:** ^1^Department of Neurophysiology, Hirosaki University Graduate School of Medicine, Hirosaki, Japan; ^2^Department of Neurophysiology, Hamamatsu University School of Medicine, Hamamatsu, Japan

**Keywords:** GABA, chloride, transporter, migration, cortex

## Abstract

During brain and spinal cord development, GABA and glycine, the inhibitory neurotransmitters, cause depolarization instead of hyperpolarization in adults. Since glycine and GABA_A_ receptors (GABA_A_Rs) are chloride (Cl^–^) ion channel receptor, the conversion of GABA/glycine actions during development is influenced by changes in the transmembrane Cl^–^ gradient, which is regulated by Cl^–^ transporters, NKCC1 (absorption) and KCC2 (expulsion). In immature neurons, inhibitory neurotransmitters are released in a non-vesicular/non-synaptic manner, transitioning to vesicular/synaptic release as the neuron matures. In other word, in immature neurons, neurotransmitters generally act tonically. Thus, the glycine/GABA system is a developmentally multimodal system that is required for neurogenesis, differentiation, migration, and synaptogenesis. The endogenous agonists for these receptors are not fully understood, we address taurine. In this review, we will discuss about the properties and function of taurine during development of neocortex. Taurine cannot be synthesized by fetuses or neonates, and is transferred from maternal blood through the placenta or maternal milk ingestion. In developing neocortex, taurine level is higher than GABA level, and taurine tonically activates GABA_A_Rs to control radial migration as a stop signal. In the marginal zone (MZ) of the developing neocortex, endogenous taurine modulates the spread of excitatory synaptic transmission, activating glycine receptors (GlyRs) as an endogenous agonist. Thus, taurine affects information processing and crucial developmental processes such as axonal growth, cell migration, and lamination in the developing cerebral cortex. Additionally, we also refer to the possible mechanism of taurine-regulating Cl^–^ homeostasis. External taurine is uptake by taurine transporter (TauT) and regulates NKCC1 and KCC2 mediated by intracellular signaling pathway, with-no-lysine kinase 1 (WNK1) and its subsequent kinases STE20/SPS1-related proline-alanine-rich protein kinase (SPAK) and oxidative stress response kinase-1 (OSR1). Through the regulation of NKCC1 and KCC2, mediated by the WNK-SPAK/OSR1 signaling pathway, taurine plays a role in maintaining Cl^–^ homeostasis during normal brain development.

## Introduction

Taurine (2-aminoethane-1-sulfonic acid), a sulfur-containing amino acid, is the most abundant amino acid in the central nervous system (CNS) and has been extensively studied (Kontro et al., 1984; Huxtable, 1989). Taurine functions as a partial activator of GABA_A_R and induces Cl^–^ currents in neuronal cells (Ye et al., 1997). Compared to the adult brain, the immature brain contains higher levels of taurine, despite the limited ability to produce taurine during fetal development (Kaczmarek, 1976; Hayes and Sturman, 1981; Stipanuk et al., 1984; Ghisolfi, 1987; Benitez-Diaz et al., 2003). In mammals, taurine, an essential nutrient for fetal development, is acquired from the maternal source via the placenta during gestation, and neonates receive taurine through maternal milk ingestion (Sturman et al., 1977; Sturman, 1981). In addition, taurine concentrations are significantly higher in umbilical venous plasma than in the maternal artery, and taurine acts as a trophic factor and neuromodulator in the development of the CNS (Bernardi, 1985; Michel et al., 1994; Cetine et al., 1996; Chen et al., 1998). In kittens with a taurine deficiency, it was reported that a delay in the migration of granule cells from the outer layer of the cerebellum to the inner layers (Sturman et al., 1985). In addition to physiological functions of taurine in the developing brain, which have been established in previous studies, we demonstrated that endogenous taurine plays a role in activating GABA_A_Rs and influencing radial migration in the developing cerebral cortex (Furukawa et al., 2014).

In the developing brain, the primary inhibitory neurotransmitter GABA elicits depolarization, while in the adult brain, it induces hyperpolarization. This switch in the effects of GABA from depolarization (resulting in Cl^–^ efflux) to hyperpolarization (resulting in Cl^–^ influx) during development is attributed to changes in the Cl^–^ gradient across the cell membrane. The regulation of this gradient involves cation-chloride cotransporters, such as NKCC1 (which facilitates Cl^–^ uptake) and KCC2 (which promotes Cl^–^ extrusion), specifically expressed in neurons. During the early stages of neuronal development, GABA is released through non-vesicular and non-synaptic mechanisms (Owens and Kriegstein, 2002; Manent et al., 2005). Consequently, the activation of GABA_A_Rs is typically tonic and primarily influenced by the ambient GABA present in the surrounding environment. In immature neurons, GABA_A_R-mediated tonic conductance is depolarizing (sometimes excitatory) because the intracellular Cl^–^ concentration is maintained high by the balance of Cl^–^ transporters (Owens et al., 1996; Ben-Ari, 2002; Yamada et al., 2004). It is believed that this tonic conductance through GABA_A_Rs plays a crucial role in various developmental processes, including neurogenesis (LoTurco et al., 1995; Haydar et al., 2000; Andang et al., 2008), neuronal migration (Behar et al., 1996, 1998, 2000, 2001; López-Bendito et al., 2003; Cuzon et al., 2006; Heck et al., 2007; Bortone and Polleux, 2009; Denter et al., 2010; Inada et al., 2011; Inoue et al., 2012), and synaptogenesis (Nakanishi et al., 2007; Wang and Kriegstein, 2008).

**FIGURE 1:**
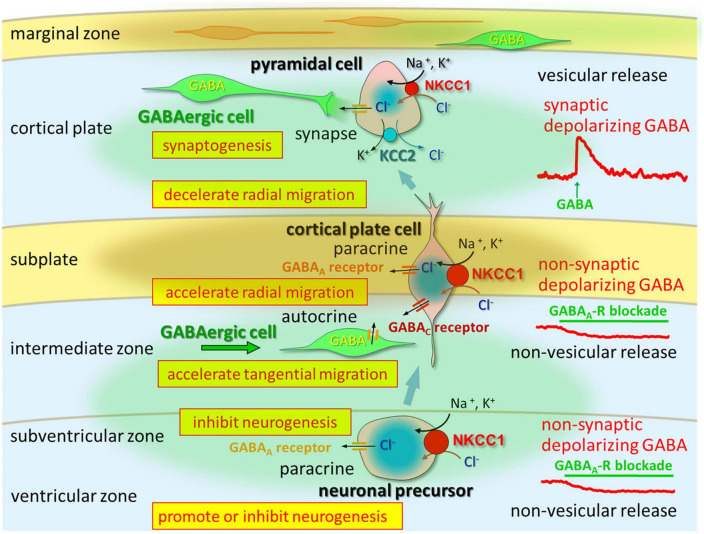
In the VZ and SVZ, ambient GABA affects neurogenesis. When post-mitotic neurons migrate to the CP, they are tonically depolarized by ambient GABA, which is released from tangentially migrating GABA neurons prior to forming synapses. Taurine taken-up and released by CP, subplate, and MZ neurons activates GABA_A_ receptors in migrating cells. Vesicular release of GABA, which remains depolarizing, could contribute to synapse formation. Following establishment of GABAergic synapses and prior to hyperpolarization, GABA acts as an excitatory neurotransmitter. Up to this stage, intracellular chloride concentration levels are high due to NKCC1 and KCC2. Following up-regulation of KCC2 and down-regulation of NKCC1, GABA acts as an inhibitory neurotransmitter. Adapted from Fukuda and Wang (2013).

Altered GABAergic functions that arise during early brain growth are caused by variations in Cl^–^ homeostasis and play important roles in the development of the neocortex by regulating processes such as synaptogenesis and laminar organization (Figure 1). During neural development, GABA, which is non-synaptically released from GABAergic neurons, has a paracrine effect on immature neurons (Demarque et al., 2002; Manent et al., 2005), which may influence both radial migration (Behar et al., 1996, 1998, 2000, 2001; Heck et al., 2007; Denter et al., 2010) and tangential migration (López-Bendito et al., 2003; Cuzon et al., 2006; Bortone and Polleux, 2009). The MZ plays a critical role in the developmental processes of cell migration and lamination within the cerebral cortex. Although Cajal-Retzius cells and non-Cajal-Retzius cells in the MZ are temporary cell populations, they both receive functional synaptic inputs and play a role in transient synaptic circuits. These synaptic interactions involving different cell types within the MZ are believed to be instrumental in the activity-dependent maturation of the neocortex, considering their vital contribution to structural development. Previous studies have highlighted the significance of these synaptic integrations in understanding the dynamic developmental processes occurring in the neocortex (Hestrin and Armstrong, 1996; Zhou and Hablitz, 1996; Radnikow et al., 2002; Luhmann et al., 2003; Soda et al., 2003). The intricate synaptic networks formed within the MZ are likely to contribute to the overall functional and structural organization of the cerebral cortex during development (Kilb et al., 2011).

The transcriptional regulation of KCC2 is influenced by brain-derived neurotrophic factors (Aguado et al., 2003; Rivera et al., 2004; Coull et al., 2005) and GABA (Ganguly et al., 2001). Additionally, KCC2 function is modulated through post-translational mechanisms such as phosphorylation (Kelsch et al., 2001; Fiumelli et al., 2005; Khirug et al., 2005; Wake et al., 2007) and other pathways (Balakrishnan et al., 2003; Ikeda et al., 2004; Blaesse et al., 2006; Inoue et al., 2006). Previous studies suggest that the WNK-SPAK/OSR1 signaling pathway plays a role in the regulation and activation of NKCC1 through phosphorylation (Xu et al., 2000; Dowd and Forbush, 2003; Richardson and Alessi, 2008; Kahle et al., 2010). de Los Heros et al. (2006), Garzon-Muvdi et al. (2007), and Rinehart et al. (2009) proposed that the WNK signaling pathway is also responsible for the activation of KCCs, such as KCC2. Activation of WNKs is triggered by osmotic stress, but the upstream signaling mechanism is currently unknown (Anselmo et al., 2006; Richardson and Alessi, 2008; Zagórska et al., 2007).

## Taurine is an agonist of both GABA_A_ and glycine receptors

It is known that taurine structurally resembles GABA and glycine and interacts with both GABA_A_R and GlyRs to induce chloride currents in neuronal cells (Linne et al., 1996; Ye et al., 1997). GlyRs are pentameric proteins composed of α and β subunits. Five glycine receptor subunits have been identified consisting of 4 alpha (1–4) and one beta subunit. During development, homomeric α2 GlyRs are abundantly expressed in neurons (Kuhse et al., 1991) and are activated by taurine with lower affinity than glycine (Le-Corronc et al., 2011). GABA_A_Rs are composed of five subunits, and nineteen distinct subunits of GABA_A_Rs (α1–6, β1–3, γ1–3, δ, ε, φ, π, and ρ1–3) have been identified. Depending on the composition of subunits, GABA_A_R subtypes have different pharmacological and electrophysiological properties (Mehta and Ticku, 1999; Sieghart and Sperk, 2002). In addition, the expression patterns of GABA_A_R subunits are entirely different during development (Laurie et al., 1992; Fritschy et al., 1994). In the prenatal and early postnatal periods, neocortical neurons primarily express the α2–5, β2/3, and γ1/2 subunits (Laurie et al., 1992). The expression patterns of subunit transcripts of α2/3 and α5 were observed throughout the developing CNS. In the germinal matrix, specifically the ventricular zone (VZ), there was a high abundance of GABA_A_R α4, β1, and γ1 subunit mRNAs. However, these subunits were not detected in the intermediate zone (IZ) at embryonic day (E) 17 and E20 in rats, indicating that proliferating cells in the germinal matrix may express these specific subunits (Ma and Barker, 1995).

In rodents, during corticogenesis, GABAergic interneurons originate from the medial ganglionic eminence (MGE) and caudal ganglionic eminence (CGE) migrate tangentially toward the cortex (Marin, 2013; Lim et al., 2018). The MGE and CGE are the primary sources of cortical interneurons in the developing nervous system. As these cells migrate from the MGE to the neocortex, their sensitivity to GABA increases (Cuzon et al., 2006). Cuzon and Yeh (2011) demonstrated that migrating neurons in the MGE and cortex exhibit distinct response profiles to specific subunits, indicating the presence of different GABA_A_R isoforms. In their study, expression profiling of GABA_A_R subunits at the mRNA level (α1–5, β1–3, γ1–3, and δ) revealed elevated mRNA expression of α1, α2, α5, γ2, and γ3 subunits in the cortex.

Since E17, the mRNA levels of β subunits have been increasing at varying rates (with β3 exhibiting the highest increase, followed by β2 and then β1), with each subunit reaching its peak expression at different times. Specifically, β1 and β2 reach their peak expression levels at postnatal day (P) 12, while β3 reaches its peak expression level between E19 and P12. Additionally, the γ1 and γ2 subunits of GABA_A_Rs are upregulated at birth as they are expressed at low levels during E14. The mRNA expression of the δ and γ3 subunits appears around P0, with δ peaking at P12 and γ3 peaking at P6 (Laurie et al., 1992).

The functional characteristics and affinity of GABA_A_R for taurine vary depending on the subunit composition. Earlier investigations have established that taurine specifically affects β2 subunit containing GABA_A_R (Bureau and Olsen, 1991; Kash et al., 2004). Recombinant studies have also indicated that the effectiveness of taurine is not determined by the type of α subunit but rather by the specific β subunit. Furthermore, δ-containing receptors exhibit stronger and more effective activation by taurine compared to γ-containing receptors (Kletke et al., 2013). Based on these findings, it can be inferred that GABA_A_Rs containing αxβ1/2δ subunits are likely to be strongly activated by taurine. Immature cortical cells express GABA_A_R subunits α2–5, β1–3, γ1/2, and δ, and their expression is regulated during development (Gambarana et al., 1991; Araki et al., 1992; Cheng et al., 2006; Peden et al., 2008). Although the specific GABA_A_R subunits expressed in radially migrating cells are not completely clear, it is plausible that these cells are responsible for the effects of taurine. Additionally, our study suggests that GABA_A_Rs are activated tonically by ambient taurine rather than by ambient GABA in the fetal cerebral cortex. Thus, in the cerebral cortex of fetal mice, taurine may be the major agonist for tonic GABA_A_R, which is expressed by the radially migrating cells (Egawa and Fukuda, 2013).

## Embryonic and fetal taurine is maternal origin

In adult mammals, taurine is synthesized in the liver through the conversion of methionine and cystine. However, due to the limited activities of taurine synthase, the synthesis of taurine is minimal in the livers and brains of human fetuses and newborn infants (Gaull et al., 1972; Zlotkin and Anderson, 1982; Sturman, 1988). Therefore, taurine is often referred to as a semi-essential amino acid (Lambert et al., 2015; Tochitani, 2017). As the ability of taurine synthesize in humans and rodents is limited, taurine deficiency is appears to be the result of reduced uptake of exogenous taurine (Miyazaki and Matsuzaki, 2014). TauT is responsible for the transport of taurine from the maternal blood to the developing brain of the embryo through the placenta (Ramamoorthy et al., 1994). In addition, fetuses consume amniotic fluid, which is abundant in taurine. Although fetal taurine levels decrease after birth, infants acquire taurine from breast milk, which contains a high concentration of taurine (Gaull, 1989; Sturman, 1993). Taurine is believed to play a role in osmoregulation and neuronal modulation through its interactions with GABA_A_Rs and GlyRs (Schmieden et al., 1992; del Olmo et al., 2000; Lambert, 2004). However, its precise role in brain development remains incompletely understood.

In TauT knockout (KO) mice, taurine level are strongly reduced in various tissues: in skeletal and heart muscle, brain, kidney, retina, and in liver (Heller-Stilb et al., 2002; Warskulat et al., 2004). TauT KO mice showed reduction of body weight gain during development and muscular endurance (Watanabe et al., 2022). Additionally, Hosoi et al. (2022) investigated the effect of taurine depletion during fetal and postnatal neocortical development on the functional properties of differentiated pyramidal neurons using TauT KO mice. They found that the depletion of taurine during development resulted in significant alteration in the firing responses of pyramidal neurons to external stimuli. These observations suggest that maternal and exogenous taurine is essential for normal development of neurons after birth.

Similar effect of taurine deficiency during perinatal period in other mammals was reported. The study using monkeys showed that infants fed taurine-free soy formula had impaired growth retardation (Hayes et al., 1980; Neuringer and Sturman, 1987). In human research, addition of taurine to formula increases fat absorption (Rigo and Senterre, 1977; Sturman, 1988). Nutritional studies of human premature infants showed that infants receiving taurine-enriched formula had more mature brain stem responses and a developmental advantage in motor function (Chesney et al., 1998). Additionally, premature infants who received breast milk showed an intelligence quotient advantage over infants who never received breast milk (Chesney et al., 1998). These evidences suggests that it is likely that there are feeding-dependent actions of taurine for normal development.

## Paracrine taurine is essential for normal fetal development

Taurine plays various biological roles including ensuring tRNA stability (Suzuki et al., 2002) and promoting retinal development (Warskulat et al., 2007; Osakada et al., 2008). However, the precise signaling pathways that utilize intracellular taurine remain uncleared. During development, GABA_A_R-mediated signaling influences neurogenesis. LoTurco et al. (1995) found that GABA depolarize cells in the VZ of rat embryonic neocortex, ant that GABA_A_R antagonist application increase DNA synthesis. Their findings revealed that the depolarizing effects of GABA were associated with a reduction in progenitor cell proliferation in the developing neocortex of rats.

During the early phase of cortical neurogenesis in the VZ of rats, GABA causes cell depolarization and reduces DNA synthesis. This suggests that the presence of endogenous GABA downregulate the cell cycle and proliferation of neocortical progenitor cells (LoTurco et al., 1995; Haydar et al., 2000). GABA exhibits similar effects on neural progenitor cell populations in the subventricular zone (SVZ), where it also acts to suppress proliferation (Haydar et al., 2000). GABA exerts a significant influence on postnatal adult neurogenesis through its depolarizing effects, and the expression of NKCC1 is critical for proliferation (Liu et al., 2005; Ge et al., 2006). Notably, robust expression of NKCC1 has been observed in the neurepithelium, including the VZ and ganglionic eminence. When KCC2 was globally overexpressed in newly fertilized zebrafish embryos, it led to a reversed Cl^–^ ion gradient, which subsequently led to hyperpolarization induced by glycine in all neurons. As a consequence, there was an observed decrease in the quantity of motoneurons and interneurons, suggesting a decline in the generation of new neurons (Reynolds et al., 2008). Hence, excitation mediated by Cl^–^ ions plays a crucial role in facilitating neurogenesis during the early stages of embryonic development. Furthermore, Andang et al. (2008) provided evidence that autocrine/paracrine signaling of GABA through GABA_A_Rs inhibits the proliferation of embryonic and peripheral neural crest stem cells.

In the neocortex, different classes of neurons, such as cortical pyramidal neurons and GABAergic interneurons, arise from distinct origins and exhibit specific migration patterns. Cortical pyramidal neurons, which are glutamatergic neurons, undergo radial migration from the VZ of the dorsal telencephalon. During this process, they migrate along the radial glial scaffolding toward the cortical plate (CP). On the other hand, GABAergic interneurons, derived from the MGE and CGE, migrate tangentially within the cerebral wall, following a different path (Marín and Rubenstein, 2001). It is possible that GABA_A_R signaling can have different effects on neurons that migrate in radial or tangential directions. About radial migration, a number of studies, including pioneering *in vitro* research by Behar et al. (1996, 1998), demonstrated that GABA plays a role in cortical cell radial migration (Luján et al., 2005; Heng et al., 2007). Behar et al. (1996) conducted a study demonstrating that the modulation of neuronal migration by GABA is highly dependent on its concentration. They found that femtomolar concentrations of GABA promote migration along a chemical gradient, known as chemotaxis, while micromolar concentrations enhance random migration, known as chemokinesis. Additionally, continuous subdural application of a GABA_A_R antagonist to block GABA_A_R activity *in vivo* led to an acceleration of radial migration and the development of abnormal cortices similar to heterotopia (Heck et al., 2007). Similarly, the application of a GABA_A_R antagonist directly into the ventricles also resulted in an accelerated radial migration, indicating that the activation of GABA_A_R acts as a signaling mechanism to halt radial migration in the cortical plate. The signaling that is induced by the action of GABA_A_Rs could include Ca^2+^ signaling. Some studies demonstrated that GABA_A_R-induced depolarization in radial migration cells produced calcium influx through voltage-gated L-type Ca^2+^ channels (Maric et al., 2001; Soria and Valdeolmillos, 2002; de Lima et al., 2009). The depolarization is attributed to the high intracellular Cl^–^ concentration, which is dependent on the activity of NKCC1. Therefore, disturbances in immature Cl^–^ ion homeostasis could lead to abnormal migration patterns.

The activation of GABA_B_ receptors in neuroblastic cells or GABA_A_Rs containing ρ-subunits promotes migration from the subventricular zone (SVZ) and IZ (Behar et al., 2000; Denter et al., 2010). GABA also plays a role in regulating the tangential migration of immature GABAergic cortical interneurons (Inada et al., 2011). Taurine has been identified as a potential candidate in this process, given its accumulation in immature neurons during cerebral cortex development (Shimada et al., 1984; Flint et al., 1998). Furthermore, certain studies suggest that taurine-deficient kittens exhibit abnormal neuronal migration in the cerebellum and cerebral cortex (Sturman et al., 1985; Palackal et al., 1986). As both radial and tangential migration are regulated via GABA_A_R, taurine may act on both radiating and tangential cell migration. Avila et al. (2013) suggested that glycine receptor activation is also involved in tangential migration. They speculated that glycine rather than taurine activates glycine receptor during neonatal period (Avila et al., 2013). Therefore, regarding cell migration in the developing neocortex, taurine regulation for tangential migration may be lower than that for radial migration.

**FIGURE 2:**
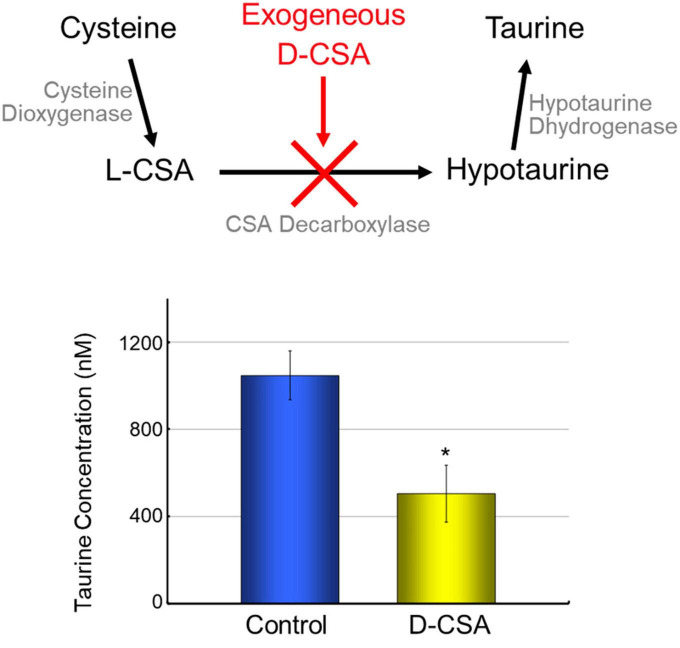
Maternal administration of D-CSA decreased ambient taurine in fetal cortex. Taurine is synthesized from cysteine via cysteine sulfinic acid (CSA) and hypotaurine. Cysteine dioxygenase and cysteine sulfinic acid decarboxylase mediates this pathway. As CSA is naturally L-type, administration of the optical isomer D-type CSA suppress taurine synthesis (Weinstein et al., 1988). The concentration of taurine released from fetal brain slices into the incubation medium was measured by HPLC, and the taurine level of brain slices from maternally D-CSA-injected fetuses was reduced by half. *P < 0.05, Student’s *t*-test.

Despite the absence of GAD65 or GAD67 expression in knockout mice, leading to a significant reduction in brain GABA levels, the structural integrity of the neocortex remains unaffected (Ji et al., 1999). This paradoxical observation prompted us to propose the existence of compensatory mechanisms that mitigate the effects of GABA deficiency. One potential solution is taurine, the dominant free amino acid during brain development, which functions as a partial activator of GABA_A_Rs (Jia et al., 2008). It was demonstrated that neuroblasts in the visual cortex of newborn kittens from taurine-depleted mothers failed to migrate and differentiate normally, indicating taurine’s critical role in regulating neuronal migration (Palackal et al., 1986). In neocortex of E17.5 mouse embryos of GAD67-GFP knock-in mice, GFP positive cells were mainly located in VZ, SVZ, and MZ. By immunohistochemical analysis, taurine signals located in MZ and SP (Furukawa et al., 2014). These results suggested that there was distinct distribution patterns of GABA and taurine in developing neocortex. When the ambient cerebral taurine concentration in homozygous embryos of GAD67-GFP knock-in mice was reduced to 50% by maternal administration of D-cysteine sulfinic acid (D-CSA), a taurine synthesis inhibitor (Figure 2), GABA_A_R-mediated tonic currents were disappeared, and radial migration of CP cells was accelerated (Furukawa et al., 2014). These findings suggest that taurine functions as an innate agonist of embryonic tonic GABA_A_R in the neocortex. Considering the distinct distribution patterns of GABA and taurine and the absence of significant differences in tonic GABA_A_R currents among the different genotypes of GAD67-GFP knock-in mice, it is plausible that maternally derived taurine acts as a stop signal for radially migrating CP cells.

In the neocortex, high expression of TauT has been detected (Smith et al., 1992). The distribution pattern of TauT in E17.5 mouse neocortex was similar to taurine distribution pattern, located in MZ and SP (Furukawa et al., 2014). TauT belong to the neurotransmitter transporter family, which relies on Na^+^ and Cl^–^ and facilitates the uptake of taurine into cells during inactive states (Tappaz, 2004). The activity of TauTs can be reversed by stimuli that disrupt the Na^+^ and Cl^–^ membrane gradient, as taurine uptake is dependent on this gradient (Oja et al., 1985; Oja and Kontro, 1987; Takuma et al., 1996). Inhibition of the TauT with the TauT inhibitor 2-(guanidino) ethanesulfonic acid (GES) led to elevated ambient taurine levels in fetal cortical slices. Furthermore, GES application during the taurine-loading phase decreased the release of taurine from cortical slices preloaded with 10 mM taurine. Additionally, GES application enhanced GABA_A_R-mediated tonic currents in subplate (SP) cells. These findings align with the expected function of TauTs in the uptake of extracellular taurine (Furukawa et al., 2014). Hence, the neocortex of fetal mice exhibits uptake of ambient taurine through the activity of TauTs, however, which types of cells in the cerebral cortex take up taurine is not yet clear.

## Endogenous taurine modulate immature state of Cl homeostasis

**FIGURE 3:**
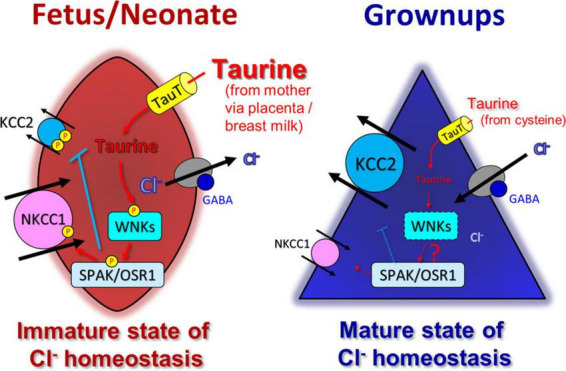
Taurine control WNK-SPAK/OSR1 signaling pathway and affect modulation of developmental switch in GABA actions induced by changes in Cl^–^ homeostasis. In immature stage, taurine, taken up into cells by TauT, activates WNK-SPAK/OSR1 pathway **(left)**. Both KCC2 and NKCC1 are phosphorylated, but their functions are oppositely regulated; KCC2 is inactivated, and NKCC1 is activated. Thus, the WNK-SPAK/OSR1 pathway maintains an immature stage of Cl^–^ homeostasis with a high intracellular chloride concentration, rendering GABA-mediated depolarization that affects neural development. In mature stage, (possibly a while after birth), the decrease in taurine reduces the activity of WNK-SPAK/OSR1 pathway, together with the upregulation and downregulation of KCC2 and NKCC1 expression, respectively, and maintains the mature stage of Cl^–^ homeostasis with a low intracellular Cl^–^ concentration **(right)**. Adapted from Fukuda and Watanabe (2019).

KCC2 activity is known to be kinase-regulated (Kelsch et al., 2001; Lee et al., 2007; Wake et al., 2007; Rinehart et al., 2009; Watanabe et al., 2009). Taurine inhibit the KCC2 activity via serine/threonine phosphorylation (Inoue et al., 2012). When residues Thr-906 and Thr-1007 residues in KCC2 were replaced by Ala (KCC2T906A/T1007A), the facilitation of KCC2 activity was observed and the inhibitory effect of taurine was prevented (Inoue et al., 2012). Exogenous taurine activates WNK1, which in turn activates downstream SPAK/OSR1. The SPAK/OSR1 kinases regulate Thr906/Thr1007 phosphorylation sites of KCC2 (de Los Heros et al., 2014; Watanabe et al., 2019). The excessive expression of active WNK1 suppresses the function of KCC2. The phosphorylation of SPAK was consistently more pronounced in embryonic brains compared to neonatal brains and was reduced by inhibiting the TauT *in vivo*. Additionally, the radial migration of the cerebral cortex was disturbed by a variant of KCC2, known as KCC2T906A/T1007A, which is insensitive to taurine and can be regulated by the WNK-SPAK/OSR1 signaling pathway. Furthermore, activation of WNK-SPAK/OSR1 pathway leads to the activation of NKCC1. Taurine induced activation of the WNK-SPAK/OSR1 pathway suppresses KCC2 and activates NKCC1, resulting in increases intracellular Cl^–^ influx and a positive shift in E_GABA_. These facts suggests that the taurine-WNK-SPAK/OSR1 signaling pathway may have a physiological role in maintaining embryonic Cl^–^ homeostasis. Thus, taurine and WNK-SPAK/OSR1 signaling may contribute to the proper maintenance of neuronal Cl^–^ homeostasis during embryonic development, which is crucial for normal brain development. Notably, the activation of WNK-SPAK/OSR1 signaling triggered by taurine may play a pivotal role in brain development (Figure 3).

In the early stages of embryonic development, several types of neurons are generated, such as Cajal-Retzius and SP cells within the cerebral cortex. These particular cells have important functions in regulating the process of cell migration within the cerebral cortex. Several studies showed that these neurons, which are generated at an early stage in the MZ and SP, were activated by GABA and glycine (Mienville, 1998; Kilb et al., 2002, 2008; Hanganu et al., 2009). These early generated neurons are capable of expressing KCC2 at the embryonic and neonatal stages (Achilles et al., 2007). In addition, as taurine is abundant in these brain regions, our findings suggest that KCC2 is dysfunctional owing to taurine distribution, affecting WNK-SPAK/OSR1 signaling and preserving GABAergic excitation. This signaling cascade may play a more extensive and crucial role in the development of the brain than previously reported.

Nugent et al. (2012) report that administering estradiol to newborn rat offspring significantly increases the levels of SPAK and OSR1 proteins, two kinases upstream of the NKCC1 cotransporter. The elevation of estradiol levels leads to a significant increase in NKCC1 phosphorylation, and this effect is reliant on transcription. Notably, the time frame of the estradiol-induced rise in NKCC1 phosphorylation coincides with that of the estradiol-induced increase in NKCC1 expression. Intriguingly, unlike taurine, estradiol does not have an impact on the protein levels of WNK kinases or pSPAK. These findings suggest that estradiol does not modulate NKCC1 signaling by exerting its effects upstream of SPAK and OSR1.

Taurine regulate WNK1 phosphorylation because WNK1S382A and WNK1S382E mutant decrease the effect of taurine on positive E_GABA_ shift (Inoue et al., 2012). The ratio of phosphor/total SPAK was downregulated at postnatal day 1 in cerebral cortex. It is suggested that WNK-SPAK/OSR1 signaling immediately decrease after birth. However, since the phosphorylated WNK1 level was not changed between embryo and postnatal rat, the phosphorylation of SPAK is not only regulated by intracellular taurine. The WNK signaling pathway can be activated by various stimuli, including osmotic stress, although the precise mechanisms underlying its activation remain unclear (Zagórska et al., 2007; Richardson and Alessi, 2008). Additionally, suppression of KCC2 function via WNK-SPAK/OSR1 signaling in rat is likely to decrease with development, however, the phosphorylation of KCC2 gradually diminishes as they progress into adulthood in mice (Rinehart et al., 2009). Further studies are necessary for the detail mechanism of WNK-SPAK/OSR1 signaling to KCC2 function.

## Ambient taurine as an endogenous agonist of tonic GABA current

The persistent activation of GABA_A_R plays a role in the tonic depolarization observed in embryonic neurons. While numerous studies have investigated the involvement of GABA_A_R activation in neocortical development, particularly in migration, they mostly relied on exogenous inhibitors (Behar et al., 1996, 1998, 2000; Bolteus and Bordey, 2004; Heck et al., 2007; Denter et al., 2010). Using a different approach from previous reports, we accessed GABA_A_R activation in the developing neocortex. In previous study, we utilized GAD67-GFP knock-in mice lacking GABA synthesis (GAD67^GFP/GFP^) and performed *in utero* electroporation to label radially migrating cells originating from the VZ. A total of 3 days after electroporation, we found normal distribution of labeled-cells even in homozygous GAD67^GFP/GFP^ mice (Furukawa et al., 2014). Furthermore, the sensitivity of labeled cells to GABA was also normal. Nonetheless, the accelerated radial migration observed in GAD67^GFP/GFP^ mice upon continuous inhibition of GABA_A_R using the GABA_A_R antagonist SR95531 suggests the involvement of alternative endogenous agonists for GABA_A_R. Therefore, ambient taurine level was focused and measured in the fetal cerebral cortex using high-performance liquid chromatography (HPLC). In E17.5 wild-type mice, the taurine concentrations released from the cerebral cortex were measured to be approximately 50 fmol/μl⋅mg, while GABA was undetectable (Furukawa et al., 2014). Taking into account the measurement capabilities of the HPLC system (GABA is detected in 0.05 fmol/μl⋅mg), it is inferred that taurine levels in the cerebral cortex of E17.5 mice are more than 1000 times higher than those of GABA (Morishima et al., 2010). Furthermore, after taurine-loading to acute slices of the cerebral cortex, the released taurine concentration increased with a 10 mM taurine-loading, but after 1 mM taurine-loading did not affect the released taurine concentration (Furukawa et al., 2014). These findings suggest that the ambient taurine concentration in the fetal cerebral cortex may reach millimolar levels. The GES increased extracellular taurine concentration by blocking taurine uptake. The elevated extracellular taurine induced a GABA_A_R-mediated tonic current. In the taurine-deficient mouse model by maternal D-CSA administration, GABA_A_R-mediated tonic currents were abolished and radial migration was accelerated. Taurine, rather than GABA, may serve as an innate activator of embryonic tonic GABA_A_R conductance, as the tonic currents induced by GABAergic excitatory stimulation were indistinguishable between GAD67-GFP knock-in mice genotypes. Taurine is likely to exert agonistic effects on tonic GABA_A_Rs, potentially functioning as a signaling mechanism to halt the radial migration of neurons. The physiological significance of the suppression of migration by taurine is not yet known, and the benefit of this suppression is also not yet known. Such points should be addressed in future studies.

Given that taurine is taken up by TauT and that GABA_A_R is activated by taurine, there would be a mechanism of taurine release to modulate GABA_A_R activation in the developing cortical cells. The exact mechanism of taurine release is still remains unknown, but several lines of evidence have been reported suggesting non-vesicular and hypo-osmotic taurine release via taurine permeable channel. Immunoelectron microscopy analysis revealed the presence of taurine within immature neurons, while it was not detected in presynaptic structures in both mice and rats (Furukawa et al., 2014; Qian et al., 2014). These findings indicate that the release of taurine might be controlled by a non-vesicular process. This discovery is consistent with earlier research proposing that taurine can be released as an osmolyte using non-vesicular mechanisms in neurons and glial cells (Calvert and Shennan, 1998; Flint et al., 1998; Mongin et al., 1999; Mulligan and MacVicar, 2006). Taurine release mediated by volume-sensitive anion channels has been documented in numerous studies (Fugelli and Thoroed, 1986; Jackson and Strange, 1993; Hall, 1995; Shennan, 1999; Haskew-Layton et al., 2008). Despite the crucial role of taurine in functional growth, there is a lack of comprehensive knowledge regarding the release of taurine in the developing nervous system, although previous studies have reported non-synaptic and hypo-osmotic taurine release in the immature rat cortex (Flint et al., 1998; Kilb et al., 2008). The utilization of HPLC in acute neocortical slices demonstrated that the introduction of 4,4′-diisothiocyanatostilbene-2,2′-di-sulfonate (DIDS), a wide-ranging Cl^–^ channel inhibitor, and 4-(2-butyl-6,7-dichlor-2-cyclopentylindan-1-on-5-yl) oxobutyric acid (DCPIB), a specific inhibitor of volume-sensitive anion channels, impeded the release of taurine. Conversely, taurine release was stimulated in a hypotonic medium (Furukawa et al., 2014). These findings provide compelling evidence that the release of taurine in the fetal cerebral cortex is facilitated through volume-sensitive anion channels (Jackson and Strange, 1993). However, it is still unclear which types of cells in the cerebral cortex release taurine, and how and when taurine release is modulated. Future studies will elucidate the detailed mechanism of taurine released in the developing cerebral cortex.

## Activity-dependent taurine release modurate network excitability

Cajal-Retzius cells are a type of early generated neurons found in the MZ of the developing rat neocortex. These cells are crucial for cell migration and lamination processes in the cerebral cortex. Previous studies have indicated the presence of excitatory GABAergic neurotransmission in the MZ, and it has been observed that Cajal-Retzius cells express NKCC1 mRNA and protein (Mienville, 1998; Kilb and Luhmann, 2001; Achilles et al., 2007). This suggests that the uptake of Cl^–^ is sufficient to maintain high intracellular Cl^–^ concentrations, which are necessary for generating excitatory responses to GABA. Disruptions in the normal regulation of Cl^–^ homeostasis and GABAergic signaling in the MZ may potentially contribute to cortical malformations, considering the MZ’s unique role in cell migration and lamination as well as its distinctive Cl^–^ homeostasis mechanisms.

Excitatory GABAergic neurotransmission, depolarization mediated by GlyRs, and the functional expression of α2/β subunits of GlyRs in Cajal-Retzius cells have been substantiated by various studies (Hestrin and Armstrong, 1996; Mienville, 1998; Kilb and Luhmann, 2001; Kilb et al., 2002; Okabe et al., 2004). The propagation of action potentials across the MZ in rats remained unaffected by the administration of glutamate receptor blockers. On the other hand, inhibition of them was observed upon the administration of either GABA_A_R or glycine receptor antagonists. Notably, the combined application of blockers targeting GABA_A_ and GlyRs resulted in the near-complete cessation of excitatory propagation. The impact of GABAA and glycine receptor antagonists on MZ neurotransmission was found to be additive, indicating the involvement of both GlyRs and GABA_A_Rs in synaptic transmission within the MZ (Qian et al., 2014). Bumetanide, an inhibitor of NKCC, has demonstrated the ability to attenuate excitation propagation in MZ. The cotransporter NKCC uptakes Cl^–^ and facilitates the accumulation of Cl^–^ in cells. Consequently, the activation of GABA_A_R can induce depolarization and occasional excitation in immature neurons. Additionally, the application of electrical stimulation to tangential slices including MZ followed by HPLC analysis revealed the release of GABA and taurine, while glycine or glutamate release was not observed (Qian et al., 2014). This suggests that the excitatory neurotransmission mediated by GABA in the rat MZ is facilitated through the activation of GlyRs by endogenous taurine. Although the release of taurine in response to depolarization and electrical stimulation has been observed in immature cortical regions, the specific mechanisms underlying activity-dependent taurine release remain unclear (Collins and Topiwala, 1974).

Several studies prove that taurine induces long-lasting enhancement of neurotransmission. In corticostriatal pathway, an involvement of taurine uptake by Na^+^-dependent TauTs accompanied by membrane depolarization has been considered (Chepkova et al., 2002, 2006; Sarkar et al., 2003; Sergeeva et al., 2003). This mechanism may accelerate the propagation of excitation, however, it is unlikely in the MZ because GES did not affect the spread of evoked signals induced by electrical stimulation. On the contrary, the induction of long-lasting synaptic transmission enhancement in the hippocampus by taurine relies on the presence of TauTs that are sensitive to GES (Galarreta et al., 1996; del Olmo et al., 2004; Dominy et al., 2004). In hippocampus, intracellular taurine accumulation rather than taurine uptake through TauT induces long-lasting synaptic potentiation (Galarreta et al., 1996; del Olmo et al., 2004; Dominy et al., 2004). While GES did not exhibit an impact on excitation within the MZ, inhibiting taurine uptake can lead to an elevation in extracellular taurine levels. It is possible that this discrepancy between the MZ and the hippocampus can be attributed to the possibility that electrical stimulation-induced extracellular taurine reaches a saturation point in facilitating excitatory propagation within the MZ. While the exact mechanism underlying the facilitatory effect of taurine on neurotransmission is yet to be fully understood, it is anticipated that taurine would augment excitatory propagation independent of GES-induced depolarization.

## The possible effect of taurine action

There are some other excitatory or facilitatory effects of taurine. In retina, taurine is the most abundant amino acid (Pasantes-Morales et al., 1972). Bulley et al. (2013) demonstrated that taurine increases the firing rate of action potentials generated by current injection in ganglion cells of retina. The taurine-induced increase of firing rate was not affected by Cl^–^ -permeable GABA and glycine receptor and GABA_B_ receptor antagonists but was suppressed by voltage-gated potassium (K_V_) channel blockers. Furthermore, endogenous inward rectifier potassium current mediated by Kv channel was reduced by 5-HT_2A_ serotonin receptor antagonist and PKC inhibitor. These results suggest that taurine facilitates neural activity by modulating serotonin system including 5-HT_2A_ receptor and PKC pathway. There is possibility that taurine increased excitation through other intracellular pathways.

In addition to the developmental regulation of K-Cl cotransporters, Cl^–^ homeostasis regulation in adult neurons has been reported. For example, the depolarizing E_GABA_ shift was induced by tetanic stimulation, epileptic activity, hyperpolarizing current pulses, synaptic activity and BDNF application (Kapur and Coulter, 1995; Avoli, 1996; Kaila et al., 1997; Rivera et al., 2002, 2004; Wardle and Poo, 2003; Woodin et al., 2003; Fiumelli et al., 2005; Wang et al., 2006). After the depolarizing E_GABA_ shift, GABA would promote neural activation and transmission. The E_GABA_ shift in adult neurons would be caused by modification of KCC2 regulation. KCC2 regulation via WNK/SPAK signaling pathway in adult was reported (Conway et al., 2017; Lee et al., 2022). The possible effect of taurine on regulation of Cl^–^ homeostasis in adults may be studied in future.

In this review, we have discussed about properties, functions, and role of maternal taurine including GABA_A_R- and GlyR-mediated actions. Otherwise, the various actions of GABA and taurine in immature neurons have been reported (Ben-Ari et al., 2007; Li et al., 2017). The GABA_A_R- and GlyR-mediated depolarization induced calcium elevation via voltage-gated calcium channels in immature neurons (Connor et al., 1987; Yuste and Katz, 1991; Owens et al., 1996). Whereas, taurine has been proposed to have antiexcitotoxic activity, and taurine diminish depolarization-induced intracellular calcium elevation by inhibition of reverse mode activity of Na^+^/Ca^2+^ exchanger in immature neuron (Zhao et al., 1999; Chen et al., 2001). This suggests that it is possible that taurine action is not simply, and other action of taurine may contribute to neural development beyond the taurine function described in this review article. Further research may reveal the detailed mechanism and novel effects of taurine on neural development.

## Conclusion and perspectives

Even though it has been established that the activity of GABA_A_Rs can promote the growth of neurons, its impact appears to differ depending on the cell type or region: activation of GABA_A_Rs can either positively or negatively regulate the proliferation of neuronal progenitors or migration of neurons. Tonic and subsequent phasic depolarization mediated by GABA_A_Rs play a crucial role in the process of synaptogenesis. During development, the intracellular Cl^–^ concentrations change, GABA_A_R containing tonically responsive subunit, and GABA and glycine receptor ligands (GABA or taurine), which is locally controlled by uptake or release mechanisms, allow the GABA_A_R-mediated actions to control the variety of developmental events in a mode and region-specific fashion. A disruption in the tonic conductance of GABA and glycine, which is regulated by the non-synaptic presence of GABA and taurine, can contribute to brain maldevelopment. Consequently, a range of pathological conditions such as epilepsy, psychiatric disorders, motor dysfunction, and neurodevelopmental disorders may be attributed, at least in part, to aberrant tonic GABA conductance. Therefore, maintaining the appropriate tone of tonic conductance and regulating the ambient GABA levels are crucial for normal brain function, and taurine, as an environmental factor acquired from the mother, is likely to play a modulatory role in CNS development.

## Author contributions

AF contributed to conception, design, and wrote the first draft of the manuscript. TF wrote sections of the manuscript. Both authors contributed to manuscript revision, read, and approved the submitted version.
